# Spurious Electrolyte and Creatinine Levels in Previously Frozen Serum Samples

**DOI:** 10.1016/j.ekir.2026.107106

**Published:** 2026-09-24

**Authors:** Kajal Kamboj, Rama Varanasi, Courtney Huynh, Mixin Lu, Anna Aceituno, Samuel Camilli, Yachana Kataria, Emmanuel Jarquín, Ramón Garcia-Trabanino, Daniel R. Brooks, Vivek Bhalla, David Friedman, Sushrut S. Waikar, CKDu CURE Consortium, Juan José Amador, Vivek Bhalla, Daniel Brooks, Sophie E. Claudel, Jennifer Crowe, Mariela Arias-Hidalgo, Lawrence S. Engel, Nora Franceschini, David Friedman, Ramón García-Trabanino, Marvin González-Quiroz, Emmanuel Jarquín, Vivekanand Jha, Bonnie Joubert, Karen Kesler, Jill Lebov, Adeera Levin, Susan Mendley, Sumit Mohan, Ana Navas-Acien, Afshin Parsa, Madeleine K. Scammell, Sushrut S. Waikar

**Affiliations:** 1 Department of Medicine, Section of Nephrology, Boston Medical Center and Boston University Chobanian & Avedisian School of Medicine, Boston, Massachusetts, USA; 2 Social Statistical and Environmental Sciences Division, RTI International, Research Triangle Park, North Carolina, USA; 3 Department of Pathology and Laboratory Medicine, Boston University Chobanian & Avedisian School of Medicine, Boston, Massachusetts, USA; 4 Agency for Agricultural Development and Health (AGDYSA), San Salvador, El Salvador; 5 Centro de Hemodialisis, San Salvador, El Salvador; 6 Department of Epidemiology, Boston University School of Public Health, Boston, Massachusetts, USA; 7 Division of Nephrology, Stanford University School of Medicine, Stanford, California, USA; 8 Renal Division, Beth Israel Deaconess Medical Center, Boston, Massachusetts, USA

## Introduction

Errors in laboratory testing can arise in 3 phases—preanalytical, analytical, and postanalytical—and can affect both scientific research and routine patient care.^1^ In the preanalytical phase, handling errors can distort results without overt indicators, unlike analytical failures that trigger immediate instrument alerts. A key vulnerability arises during the thawing of frozen serum or plasma samples—the freezing process generates vertical concentration gradients within the tube that must be abolished by thorough mixing before aliquoting and analysis. Several reports in the clinical and research literature have identified inadequate mixing of thawed samples as a likely cause of spurious hyponatremia.^2^, ^3^, ^4^, ^5^

As part of a planned quality assurance effort, we conducted a pilot analysis of frozen serum samples from 64 participants in the international, multicenter Chronic Kidney Disease of Uncertain Etiology in Agricultural Communities (CURE) Research Consortium study.^6^ In this pilot study designed to identify potential analytical errors, we discovered implausible rates of severe electrolyte abnormalities. A comprehensive review of preanalytical, analytical, and postanalytical sources of error identified a vulnerability in the standardized processing workflow —to maintain a uniform protocol across geographically dispersed sites, processed samples were stored in 5 ml cryovials that required secondary aliquoting at the US central biorepository. The CURE study manual of operations outlined all details of sample collection and sample processing from field epidemiology sites and at the central biorepository (included in Supplementary methods). We noted that the manual of operations required mixing thawed serum samples with a vortex mixer, which is frequently used in research laboratories to mix small-volume aliquots. This instrument achieves rapid homogenization by imparting high-frequency orbital motion to the tube, creating a turbulent vortex to mix the liquid. We suspected that vortexing 5 ml, round-bottomed cryovials instead of manual inversion might have led to the spurious electrolyte values through inadequate mixing. To test this hypothesis, we conducted a pilot study in serum samples collected from patients in a nephrology clinic at a Boston hospital. We simulated the preanalytical conditions from the CURE study, using the following 3 mixing conditions: manual inversion ×2, manual inversion ×10, and vortexing ×5 seconds. We also created an aqueous saline solution approximating normal human Na^+^ and K^+^ concentrations as an additional test sample (detailed in the Supplementary methods). Here, we report the experimental confirmation of this hypothesis.

A full protocol is provided in the Supplementary Methods.

## Results

The pilot CURE consortium study of 64 participants identified implausible electrolyte abnormalities (median serum sodium 116 mmol/l; median potassium 3.0 mmol/l; Supplementary Table S1). In our experimental samples, we similarly observed significant electrolyte abnormalities in thawed samples that underwent vortexing, with a systematic gradient by depth, in which low measured values were at the top of the tube and high values at the bottom (Table 1).

**Table 1:** Concentration of analytes in samples subjected to various processing methods

Sample	Mixing method	Initial freezing condition	Sodium (mmol/l)				Potassium (mmol/l)				Chloride (mmol/l)				Calcium (mg/dl)				Creatinine (mg/dl)			
			Clinical lab value	T	M	B	Clinical lab value	T	M	B	Clinical lab value	T	M	B	Clinical lab value	T	M	B	Clinical lab value	T	M	B
Patient 1	Inversion x 10 times	−80°	137	135	136	134	4.1	4.2	4.2	4.2	103	101	101	100	9.5	8.9	9.0	8.8	1.28	1.21	1.19	1.18
	Vortex x 5 s	−80°	137	105	134	161	4.1	3.3	4.1	4.9	103	79	99	119	9.5	6.7	8.8	11.1	1.28	0.80	1.17	1.50
Patient 2	Inversion x 2 times	−80°	140	137	138	138	4.2	4.0	4.1	4.0	98	97	98	98	10.4	9.9	10	10	1.19	1.18	1.17	1.17
	Vortex x 5 s	−80°	140	105	145	167	4.2	3.2	4.4	5.1	98	75	103	117	10.4	7.3	10.5	12.5	1.19	0.77	1.25	1.42
Patient 3	Inversion x 10 times	−80°	137	135	138	137	4.6	4.5	4.5	4.5	104	104	106	105	9.3	8.6	8.8	8.7	2.41	2.31	2.38	2.36
	Vortex x 5 s	−80°	137	109	140	165	4.6	3.6	4.6	5.4	104	84	106	125	9.3	6.8	9.0	10.9	2.41	1.73	2.41	2.95
Patient 4	Inversion x 10 times	−20 °C to −80 °C	142	139	138	139	4.1	4.2	4.2	4.2	105	104	104	104	9.9	9.3	9.2	9.4	1.86	1.87	1.82	1.84
	Vortex x 5 s	−20 °C to −80 °C	142	103	142	170	4.1	3.1	4.2	5.0	105	78	106	126	9.9	6.6	9.4	11.7	1.86	1.26	1.86	2.35
Saline solution	Inversion x 10 times	−20 °C to −80 °C	-	131	131	131	-	3.2	3.2	3.2	-	133	133	133	-	-	-	-	-	-	-	-
	Vortex x 5 s	−20 °C to −80 °C	-	102	133	167	-	2.5	3.3	4.0	-	104	136	>150	-	-	-	-	-	-	-	-

Serum samples from 4 patients and saline solution were thawed overnight and handled either by inversion or vortex as indicated. Samples were then aliquoted from top (T), middle (M) and bottom (B) and were analysed. Clinical lab values indicate the biochemistry analysis concentrations on the day of enrolment.

Reference Na^+^ concentrations measured in the hospital’s clinical chemistry laboratory averaged 139 mmol/L (range: 137–142 mmol/l) across the 4 patient samples. By contrast, the average Na^+^ measured at the top and bottom of vortexed samples was 105 mmol/l (range: 103–109 mmol/l) and 166 mmol/l (range: 161–170 mmol/l), respectively. Saline solution results were consistent—top 102 mmol/l, middle 133 mmol/l, and bottom 167 mmol/l in vortexed samples. Inverted samples did not demonstrate concentration gradients. Mixed-effects regression confirmed that no statistically significant difference in analyte concentration by depth was observed with inversion, whereas all analytes showed significant differences after vortexing (Table 2).

**Table 2:** Mean change in serum analyte concentration per incremental depth level: top, middle, bottom

Parameter	Vortex depth gradient (95% CI)	Inversion depth gradient (95% CI)
Sodium (mmol/l)	30.1 (25.7–34.6)	0.3 (−0.9 to 1.4)
Potassium (mmol/l)	0.9 (0.8–1.0)	0 (−0.04 to 0.04)
Chloride (mmol/l)	21.4 (18.1–24.6)	0.1 (−0.6 to 0.8)
Calcium (mg/dl)	2.4 (2.1–2.7)	0.02 (−0.1 to 0.1)
Creatinine (mg/dl)	0.5 (0.3–0.6)	0 (−0.03 to 0.02)
Blood urea nitrogen (mg/dl)	6.6 (4.7–8.6)	0 (−0.5 to 0.5)
Uric acid (mg/dl)	1.6 (1.3–1.8)	0.04 (−0.04 to 0.1)

Results are presented as mean difference (95% confidence interval), estimated from linear mixed-effects models.

Depth Gradient: difference between consecutive depth levels (top→middle, middle→bottom).

## Discussion

We identified and experimentally confirmed that vortexing—rather than manually inverting—thawed 5 ml serum cryovials was the cause of spurious electrolyte values during the planned quality assurance analysis of the CURE consortium pilot study. The magnitude of the gradients observed (top-to-bottom Na^+^ spanning ∼ 60 mmol/l in a single tube) underscores the clinical importance of proper mixing after thawing. Subsequent analysis of additional clinical samples helped elucidate the source of the identified errors, highlighting the importance of appropriate mixing of thawed serum samples.^3^

Vertical concentration gradients form during freezing through 2 physical processes, as described previously.^7^ First, as water freezes, it forms pure ice crystals that exclude dissolved solutes (Na^+^, K^+^, creatinine, proteins), concentrating them in the remaining unfrozen liquid. Second, freezing is not spatially uniform—a cryovial cools from the outside inward, generating density-driven convection currents. Because water reaches its maximum density at 4 °C rather than at 0 °C, the colder, denser water near the tube walls sinks toward the bottom whereas warmer water rises through the center. This convective circulation continues through an increasingly narrow unfrozen channel as ice formation progresses, continuously driving solutes downward. Dissolved solutes further depress the freezing point of the liquid at the bottom, so the bottom of the tube is the last region to freeze and the most concentrated. The result is a steep vertical gradient, with solute-depleted ice at the top and a concentrated solute-rich liquid at the bottom. On thawing, this gradient persists. Vortexing imparts high-frequency orbital (centrifugal) motion to the liquid, creating radial mixing within a horizontal plane but providing little axial (top-to-bottom) fluid exchange. Manual inversion, by contrast, displaces the full column of liquid end-to-end, abolishing the concentration gradient. Our data demonstrate this directly—inversion eliminated the gradient entirely, whereas vortexing did not.

These findings are consistent with previous reports. Ekström *et al.* demonstrated analyte gradients in thawed samples that were unmixed or vortexed for 2 to 3 seconds, and showed that only inversion-based mixing sufficiently abolished them.^3^ Marquez *et al.*^4^ reported critically low serum sodium values attributable to unrecognized sample freezing during shipment from a remote clinic to a central laboratory, a scenario that is identical to ours.

Vortexing is standard in basic science labs for mixing small-volume aliquots where manual inversion is impractical, as surface tension enables efficient mixing with minimal shear. This convention led us to specify vortexing in the CURE manual of operations, without recognizing that it is inadequate for large-volumes such as 5 ml cryovials, where full-column mixing is required and vortexing may introduce greater shear than gentle inversion—an effect likely amplified in tall, narrow 5 ml cryovials, where the aspect ratio exaggerates the vertical gradient.

Unlike routine blood collection procedures, where tube inversion is primarily intended to facilitate appropriate mixing with collection-tube additives, technicians in research settings processing frozen human biospecimens may be less familiar with the importance of adequate mixing. This issue extends beyond Chronic Kidney Disease of Uncertain Etiology research. Based on our results, we recommend thorough manual inversion of thawed samples in large-volume cryovials before subaliquoting rather than relying on vortexing alone. Any study or biorepository that freezes serum or plasma in large-volume cryovials and subsequently subaliquots after thawing is vulnerable to this error. Several Chronic Kidney Disease of Uncertain Etiology studies have reported hyponatremia and hypokalemia as prevalent electrolyte findings. Although detailed information regarding sample collection and preanalytical handling is not available for all studies, some studies were performed using previously frozen samples (See the Supplementary ReferencesS1–S6 and Supplementary Table S2), raising the possibility that spurious gradients may have contributed to reported electrolyte abnormalities. Based on our results, we recommend that thawed samples in large-volume cryovials to be mixed by manual inversions rather than vortexing before subaliquoting. Improper or insufficient mixing may not be readily apparent once samples reach the laboratory; laboratory protocols and manuals of operations therefore should specify preanalytical details such as mixing techniques explicitly during study design. Our findings specifically apply to the experimental conditions evaluated, that is, use of large-volume serum aliquots, and may not necessarily be generalizable to serum that is separated and aliquoted into smaller volumes immediately after collection, as this condition was not evaluated in the current study.

Our findings serve as an important cautionary example for future consortia relying on centralized biorepository infrastructure. As a result, the CURE central biorepository revised their procedures and retrained staff to perform 10 inversions for mixing of thawed samples in cryovials. Measurements from samples described in this study will be excluded from CURE reports and replaced with measurements on appropriately handled samples.

## Appendix

### List of Members of the Chronic Kidney Disease of UnceRtain Etiology (CKDu) in Agricultural Communities (CURE) Research Consortium

Juan José Amador, Vivek Bhalla, Daniel Brooks, Sophie E. Claudel, Jennifer Crowe, Mariela Arias-Hidalgo, Lawrence S. Engel, Nora Franceschini, David Friedman, Ramón García-Trabanino, Marvin González-Quiroz, Emmanuel Jarquín, Vivekanand Jha, Bonnie Joubert, Karen Kesler, Jill Lebov, Adeera Levin, Susan Mendley, Sumit Mohan, Ana Navas-Acien, Afshin Parsa, Madeleine K. Scammell, and Sushrut S. Waikar.

## Disclosure

VJ reports consulting fees/honoraria from Bayer, Boehringer Ingelheim, Vantive, Vera, Otsuka, Novartis, AstraZeneca, Timberlyne, and Biogen under the policy of all payments going to the George Institute. SSW reports consulting fees from Bain, CANbridge, Dechert, Delix, Finnegan, Ikena, Merck, Mineralys, Motric Bio, Ono Pharma, PepGen, Quinn, Ropes & Gray, Strataca, Tucker-Ellis, Vertex, and Wolters Kluwer. VB reports consulting fees from AstraZeneca, Idorsia, Medtronic, and Nephrogen, and ownership interest in Nephrogen. VJ reports consulting fees/honoraria from Bayer, Boehringer Ingelheim, Vantive, Vera, Otsuka, Novartis, AstraZeneca, Timberlyne, and Biogen under the policy of all payments going to the George Institute. All other authors declared no competing interests.

## Supplementary Material

Supplementary File (PDF) **Supplementary Methods. Supplementary References. Table S1.** Distribution of electrolyte values in pilot samples from participants in the Chronic Kidney Disease of Uncertain Etiology in Agricultural Communities (CURE) Research Consortium (N = 64). **Table S2.** Reported prevalence of hyponatremia and hypokalemia in CKDu/MeN studies.

## Footnotes

Supplementary File (PDF)
**Supplementary Methods**. **Supplementary References**. **Table S1.** Distribution of electrolyte values in pilot samples from participants in the Chronic Kidney Disease of Uncertain Etiology in Agricultural Communities (CURE) Research Consortium (N = 64). **Table S2.** Reported prevalence of hyponatremia and hypokalemia in CKDu/MeN studies.

## References

[1] Nordin N., Ab Rahim S.N., Wan Omar W.F.A. (2024). Preanalytical errors in Clinical Laboratory testing at a glance: source and control measures. Cureus.

[2] Chang C.E. (1983). Segregation of proteins and sodium in human plasma upon freezing. Vox Sang.

[3] Ekstrom U., Apelqvist J., Hansson E. (2020). Insufficient mixing of thawed serum samples leading to erroneous results - experience from a field study and use of a correction procedure. Scand J Clin Lab Invest.

[4] Marquez C.P., Petersen J.R., Okorodudu A.O. (2018). Critically low sodium levels due to concentration gradients formed in patient samples after undergoing a freeze-thaw cycle. Clin Chim Acta.

[5] Omang S.H., Vellar O.D. (1973). Analytical error due to concentration gradients in frozen and thawed samples. Clin Chim Acta.

[6] Lebov J.F., Brooks D.R., Aceituno A. (2025). Rationale and design of the international prospective study of CKD of uncertain etiology in agricultural communities. Kidney Int Rep.

[7] Rodrigues M.A., Miller M.A., Glass M.A., Singh S.K., Johnston K.P. (2011). Effect of freezing rate and dendritic ice formation on concentration profiles of proteins frozen in cylindrical vessels. J Pharm Sci.

