# Supplementary material for: Spurious Electrolyte and Creatinine Levels in Previously Frozen Serum Samples
Source: Kidney Int Rep. 2026 Sep 24;11(12):107106. doi: 10.1016/j.ekir.2026.107106 (PMC13697213; doi:10.1016/j.ekir.2026.107106)
Supplement: Supplementary file 1 [file mmc1.pdf]

Supplementary Materials

Supplementary Methods:

Manual of operations (MOP): The initial CURE study MOP outlined the following steps for field sites to collect serum for blood chemistries: venipuncture into 10 mL SST serum tubes, inversion x 8-10, upright storage at ambient temperature for 30-120 minutes, refrigeration in a cooler at 2-8 °C, and then transport to a local laboratory for additional processing. The MOP outlined the following steps for sample processing at the laboratory: centrifugation of serum tubes at 2000 RCF for 12 minutes in a refrigerated centrifuge; removal of supernatant using a 1000 uL pipette tip into a 5 mL cryovial can store up to 4.5 ml serum (Cat. Simport-T310-5A); and then storage of the 5 mL cryovial at -20 °C or -80 °C. Samples initially frozen at -20 °C were eventually transferred to a -80 °C freezer within 3 weeks and shipped on dry ice every 1-2 months to a biorepository in North Carolina, with temperature loggers to confirm cold chain of custody. At the biorepository, samples were thawed overnight at 2-8°C, vortexed x 5 seconds, and then subaliquoted into 10 child aliquots. Single 500 uL aliquots from each of 64 pilot study participants were then sent overnight on dry ice to a CLIA-certified clinical laboratory in Boston for measurement of electrolytes, BUN, creatinine, cystatin C, and uric acid.

Patient recruitment: We obtained written informed consent to obtain two additional blood tubes from four patients in the Nephrology clinic who were having chemistries measured for clinical purposes. Two 10mL tiger top SST tubes (Cat. BD-367988) per patient were withdrawn, allowed to clot at room temp for 30-60 minutes followed by centrifugation at 2000 g for 12 min at 4°C. The serum was then placed into two labelled 5 mL tubes. To reflect normal plasma K^+ and Na^+ levels without other blood components, we also created an aqueous salt solution with 4.3 mEq KCl and 140 mEq NaCl. We then subjected samples to a variety of

conditions to isolate the potential effects of freezing temperature. 3 patients had their 5mL serum samples immediately transferred to -80°C. The remaining 1 patient had their serum samples frozen at -20°C for 4 days and then transferred to -80°C to replicate repository storage conditions and evaluate the impact of varied temperature environments. The saline sample was also frozen at -20°C for 4 days and then transferred to -80°C.

A week later, the samples were thawed overnight at 2-8 °C and then subjected to simulated conditions for mixing: vortex for 5 seconds on a vortex shaker (Fisher Scientific Vortex-Genie 2 at high-speed intensity of 8, ~3000 rpm) versus manual inversion x10 in 3 patients and x2 for 1 patient.

After this procedure, 1.3mL aliquots of serum were manually taken by pipette from the top (T), middle (M) and bottom (B) from the tubes into cryovials. To evaluate vertical solute stratification, 1.3 mL aliquots were aspirated from a 5 mL serum column (approximate height 60 mm) contained within 90 mm self-standing cryovials. Aspirations were performed using extended-length 1000 µL pipette tips (100 mm) to ensure precise depth control. The **'Top'** aliquot was taken from the upper 5 mm of the meniscus, the **'Middle'** aliquot from the 30 mm depth, and the **'Bottom'** aliquot from the final 5 mm at the base of the vial. This sampling strategy ensured that each 1.3 mL fraction represented a distinct vertical stratum of the thawed volume. These aliquots were then frozen in a -80 °C freezer and sent the next day to the Boston Medical Center clinical chemistry laboratory for measurement of a basic chemistry panel. At the clinical lab, samples were handled identically by thawing and then mixing by inversion x 10. The saline sample underwent chemical analysis for Na^+, K^+, and Cl^-. All study measurements were made in a CLIA certified laboratory and compared against clinical results on the same blood draw from the hospital laboratory. Abbott™ Alinity™ c analytical system (Abbott Laboratories, Abbott Park, Illinois, US) chemistry analyzer was used for biochemistry analysis.

Statistical analysis: Linear mixed effects regression models with heterogeneous residual variances across procedures was used for the analysis of analytes. Outcome measures were the difference between measured and true concentrations of analytes. Procedure (inversion vs. vortex), depth i.e. top, middle, bottom (treated as a continuous variable), and their interaction (procedure × depth) were taken as covariates. R software was used for statistical analysis.

Supplementary Table S1: Reported prevalence of hyponatremia and hypokalemia in CKDu/MeN studies

**Author (year)**	**Design of study/ Patients**	**No. of Participants**	**Were samples frozen before analysis?**	**Prevalence of hyponatremia/hypokalemia**
Valdes *et al.* (2015)[S1]	Descriptive study; CKDu women of agricultural communities in El Salvador	N=10	No details provided	Hyponatremia in 60% (<136mEq/L))<br>Hypocalcemia in 50% (<8.5 mg/dL)<br>Hypokalemia in none (<3.5 mEq/L)
Wijkstrom *et al.* (2017)[S2]	Prospective cross-sectional study; MeN men of sugarcane fields in Nicargua/<br>Follow up of agricultural workers at El Salvador with MeN	N=19; N=8 at baseline<br><br>N=18; N=7 at follow up	Yes (frozen at -20°C for 4 days, then shipped followed by storage at -70°C)	Kidney biopsy study at Nicargua:<br>· Hyponatremia in 47% at baseline (<137 mEq/L)<br>· Hypokalemia in 21% at baseline (<3.5 mEq/L)<br>· Hyponatremia in 22 % at follow up (<137 mEq/L)<br>· Hypokalemia in 11% at follow up (<3.5 mEq/L)<br>Kidney biopsy study at El Salvador:<br>· Hyponatremia in 57% at baseline (<137 mEq/L)<br>· Hypokalemia in 28% at follow up (<3.5 mEq/L)
Ekstrom *et al.* (2020)[S3]	Cross-sectional study; CKDu subjects from Central America (sugar mill workers of El Salvador and Nicargua)	N=352	Yes (frozen at -20°C for 4 days, then shipped followed by storage at -80 °C)	Hyponatremia in 14% (<115 mmol/L)
Frenando *et al.* (2020)[S4]	Cross-sectional, CKDu subjects from Sri Lanka	N=132	No: samples were assayed the day of collection	Hyponatremia in 23.5% (<136 mEq/L)<br>Hypokalemia in 5% (<3.5 mEq/L)
Parida *et al.* (2022)[S5]	Cross-sectional; CKDu subjects from India	N=23	No details provided	Hyponatremia in 95.6% (<135 mEq/L)<br>Hypokalemia in 47.8% (<3.5 mEq/L)
Galhotra *et al.* (2024)[S6]	Prospective and Retrospective cross-sectional study; CKDu subjects from Central India	N=166	No: samples were assayed the day of collection	Hyponatremia in 36.7% (<120 mmol/L-3%, 120-130 mmol/L-15.6%, 130-135 mmol/L-18.7%)<br>Hypokalemia in 12% (<3.5 mmol/L-12%)

Abbreviations: CKDu, chronic kidney disease of uncertain etiology; MeN, Mesoamerican nephropathy

Supplementary Table S2: Distribution of electrolyte values in pilot samples from participants in the Chronic Kidney Disease of UnceRtain Etiology in Agricultural Communities (CURE) Research Consortium (N=64)

Analyte	Critically low	Low	Normal	High
**Sodium (mmol/L)**	<110	110–125	126–145	>145
**N (%)**	19 (30%)	29 (45%)	14 (22%)	2 (3%)
**Potassium (mmol/L)**	<2.5	2.5–3.4	3.5–5.0	>5.0
**N (%)**	3 (5%)	30 (47%)	31 (48%)	0 (0%)
**Chloride (mmol/L)**	<86	86–95	96–106	>106
**N (%)**	25 (39%)	23 (36%)	14 (22%)	2 (3%)
**Calcium (mg/dL)**	<7.0	7.0–8.4	8.5–9.5	>9.5
**N (%)**	11 (17%)	34 (53%)	16 (25%)	3 (5%)

Data presented as number (percentage)

Supplementary References:

S1. Herrera Valdes R, Orantes CM, Almaguer Lopez M*, et al.* Clinical characteristics of chronic kidney disease of non-traditional causes in women of agricultural communities in El Salvador. *Clin Nephrol* 2015; **83:** 56-63.

S2. Wijkstrom J, Gonzalez-Quiroz M, Hernandez M*, et al.* Renal Morphology, Clinical Findings, and Progression Rate in Mesoamerican Nephropathy. *Am J Kidney Dis* 2017; **69:** 626-636.

S3. Ekstrom U, Apelqvist J, Hansson E*, et al.* Insufficient mixing of thawed serum samples leading to erroneous results - experience from a field study and use of a correction procedure. *Scand J Clin Lab Invest* 2020; **80:** 99-105.

S4. Fernando B, Sudeshika TSH, Hettiarachchi TW*, et al.* Evaluation of biochemical profile of Chronic Kidney Disease of uncertain etiology in Sri Lanka. *PLoS One* 2020; **15:** e0232522.

S5. Parida S, Das S, Kar A*, et al.* Clinicopathological Study of Chronic Kidney Disease of Unknown Etiology in Odisha. *J Assoc Physicians India* 2022; **70:** 11-12.

S6. Galhotra A, Rathore V, Pal R*, et al.* Clinico-Epidemiological Profile of Patients with Chronic Kidney Diseases of Unknown Etiology: A Hospital-Based, Cross-Sectional Study from Central India. *Indian J Nephrol* 2024; **34:** 241-245.
